# COVID-19: Factors associated with psychological distress, fear, and coping strategies among community members across 17 countries

**DOI:** 10.1186/s12992-021-00768-3

**Published:** 2021-10-01

**Authors:** Muhammad Aziz Rahman, Sheikh Mohammed Shariful Islam, Patraporn Tungpunkom, Farhana Sultana, Sheikh M. Alif, Biswajit Banik, Masudus Salehin, Bindu Joseph, Louisa Lam, Mimmie Claudine Watts, Sabria Jihan Khan, Sherief Ghozy, Sek Ying Chair, Wai Tong Chien, Carlos Schönfeldt-Lecuona, Nashwa El-Khazragy, Ilias Mahmud, Adhra Hilal Al Mawali, Turkiya Saleh Al Maskari, Rayan Jafnan Alharbi, Amr Hamza, Mohamad Ali Keblawi, Majeda Hammoud, Asmaa M. Elaidy, Agus Dwi Susanto, Ahmed Suparno Bahar Moni, Alaa Ashraf AlQurashi, Almajdoub Ali, Amit Wazib, Cattaliya Siripattarakul Sanluang, Deena H. Elsori, Farhana Yasmin, Feni Fitrani Taufik, Manal Al Kloub, Mara Gerbabe Ruiz, Mohamed Elsayed, Nael Kamel Eltewacy, Nahed Al Laham, Natalia Oli, Ramy Abdelnaby, Rania Dweik, Ratree Thongyu, Sami Almustanyir, Shaila Rahman, Sirirat Nitayawan, Sondos Al-Madhoun, Suwit Inthong, Talal Ali Alharbi, Tamanna Bahar, Tribowo Tuahta Ginting, Wendy M. Cross

**Affiliations:** 1 School of Health, Federation University Australia, Berwick, Victoria Australia; 2 Australia Institute for Primary Care and Ageing (AIPCA), La Trobe University, Melbourne, Victoria Australia; 3 Department of Noncommunicable Diseases, Bangladesh University of Health Sciences (BUHS), Dhaka, Bangladesh; 4 Deakin University, Burwood, Victoria Australia; 5 Faculty of Nursing, Chiang Mai University, Chiang Mai, Thailand; 6 Telstra Health, Melbourne, Victoria Australia; 7 Monash University, Clayton, Victoria Australia; 8 The University of Melbourne, Melbourne, Victoria Australia; 9 Neurovascular Research Lab, Radiology Department, Mayo Clinic, Rochester, MN USA; 10 The Nethersole School of Nursing, The Chinese University of Hong Kong, Shatin, New Territories Hong Kong; 11 Psychiatric University Clinic Ulm, Ulm, Germany; 12 Faculty of Medicine, Ain Shams University, Cairo, Egypt; 13 Department of Public Health, College of Public Health and Health Informatics, Qassim University, Al Bukairiyah, Kingdom of Saudi Arabia; 14 Ministry of Health, Muscat, Sultanate of Oman; 15 Oman College of Health Sciences-South Sharquiya, Sur, Sultanate of Oman; 16 Department of Emergency Medical Service, Jazan University, Jazan, Kingdom of Saudi Arabia; 17 Faculty of Medicine, University of Aleppo, Aleppo, Syria; 18 Faculty of Medicine, Kuwait University, Kuwait City, Kuwait; 19 Faculty of Medicine for Girls, Al-azhar University, Cairo, Egypt; 20 Department of Pulmonology and Respiratory Medicine, Universitas Indonesia, Jakarta, Indonesia; 21 Advanced Medical and Dental Institute, Universiti Sains Malaysia, Penang, Malaysia; 22 King Fahad Medical City, Riyadh, Kingdom of Saudi Arabia; 23 Brega General Hospital, Brega, Libya; 24 Enam Medical College & Hospital, Dhaka, Bangladesh; 25 Abu Dhabi University, Abu Dhabi, United Arab Emirates; 26 Lahore Garrison University, Lahore, Pakistan; 27 The Hashemite University, Zarqa, Jordan; 28 Department of Psychiatry and Psychotherapy, University of Ulm, Ulm, Germany; 29 Faculty of Pharmacy, Beni-Suef University, Minia, Egypt; 30 Al Azhar University-Gaza, Gaza strip, Palestine; 31 Kathmandu Medical College, Kathmandu, Nepal; 32 Department of Neurology, RWTH Aachen University, Aachen, Germany; 33 Rangsit University, Pathum Thani, Thailand; 34 Ministry of Health, Riyadh, Kingdom of Saudi Arabia; 35 King Saud University Medical City, King Saud University, Riyadh, Kingdom of Saudi Arabia; 36 National Institute of Cancer Research and Hospital, Dhaka, Bangladesh; 37 Psychiatric Medical Staff Group, Persahabatan General Hospital, Jakarta, Indonesia

**Keywords:** COVID-19, coronavirus, mental health, psychological distress, fear, coping, resilience

## Abstract

**Background:**

The current pandemic of COVID-19 impacted the psychological wellbeing of populations globally.

**Objectives:**

We aimed to examine the extent and identify factors associated with psychological distress, fear of COVID-19 and coping.

**Methods:**

We conducted a cross-sectional study across 17 countries during Jun-2020 to Jan-2021. Levels of psychological distress (Kessler Psychological Distress Scale), fear of COVID-19 (Fear of COVID-19 Scale), and coping (Brief Resilient Coping Scale) were assessed.

**Results:**

A total of 8,559 people participated; mean age (±SD) was 33(±13) years, 64% were females and 40% self-identified as frontline workers. More than two-thirds (69%) experienced moderate-to-very high levels of psychological distress, which was 46% in Thailand and 91% in Egypt. A quarter (24%) had high levels of fear of COVID-19, which was as low as 9% in Libya and as high as 38% in Bangladesh. More than half (57%) exhibited medium to high resilient coping; the lowest prevalence (3%) was reported in Australia and the highest (72%) in Syria. Being female (AOR 1.31 [95% CIs 1.09-1.57]), perceived distress due to change of employment status (1.56 [1.29-1.90]), comorbidity with mental health conditions (3.02 [1.20-7.60]) were associated with higher levels of psychological distress and fear. Doctors had higher psychological distress (1.43 [1.04-1.97]), but low levels of fear of COVID-19 (0.55 [0.41-0.76]); nurses had medium to high resilient coping (1.30 [1.03-1.65]).

**Conclusions:**

The extent of psychological distress, fear of COVID-19 and coping varied by country; however, we identified few higher risk groups who were more vulnerable than others. There is an urgent need to prioritise health and well-being of those people through well-designed intervention that may need to be tailored to meet country specific requirements.

**Supplementary Information:**

The online version contains supplementary material available at 10.1186/s12992-021-00768-3.

## Introduction

The COVID-19 pandemic, with more than 226 million cases and 4.7 million deaths by mid Sep-2021, has occurred in waves [1]. The first wave raised the alarm of what was imminent; the second wave identified the in-country differences in incidence, prevalence and mortality rates as well as health system gaps, notwithstanding policy failures; while the third wave further exposed varying social, financial, policy and failures in the health system management on the global scale.

COVID-19 impacted psychological wellbeing of global populations. Studies revealed that COVID-19 pandemic affected people in discrete ways across the world and exposed varying degrees of vulnerability among divergent community members. Evidence linked emotional stress to disasters, quarantine and lockdown, where people in uncertain situations used to lose the power to predict and control their lives under conditions of threat [2]. Prevalence of psychological distress, anxiety and depression during the COVID-19 pandemic was reported as 50%, 27% and 28% respectively, in a systematic review with 398,771 participants [3]. Psychological distress had been shown to be more prevalent among middle-aged single women and mothers, and those in lower-income groups [4]. A recent review of the psychological effects of COVID-19 related lockdown reported many negative psychological effects associated with quarantine including fear, stress, insomnia, depression, frustration, and anger and some of those persisted post quarantine period [5].

Factors associated with psychological wellbeing during the current COVID-19 pandemic were diverse. However, the primary reasons for COVID-related stress were associated with contracting the virus, related complications, restrictions and mandated lockdowns, social isolation, financial loss, lack of income and disruption of daily routines which have been observed globally [6]. Moreover, critical incidents such as deaths of family members, pre-existing stressors, being older and migrant were substantial grounds for poor mental health outcomes [7]. An international study of 18 countries examining the mental health outcomes related to mandatory lockdowns showed that half of the study population (n=9,565) expressed moderate mental wellbeing; financial impacts along with lack of access to basic needs were identified as substantial grounds for such poor mental health outcomes [8]. A recent Australian study also found that people with higher psychological distress increased smoking and alcohol consumption during the pandemic period; females and people with pre-existing mental health conditions were more likely to experience higher levels of psychological distress [9]. Furthermore, being on the frontline, health care workers also confronted physical and mental health consequences of COVID 19 crisis [10].

COVID-19 was unpredictable. Varying degrees of lockdown or isolation measures were implemented nationally, depending on the stage of the pandemic. Most of the published studies examined psychological impacts of COVID-19 in a single country or small communities. A recent systematic review and meta-analysis showed that Black and Asian ethnic community people were at increased risk of COVID-19 infection, intensive care admission and deaths [11]. Evidence from multicultural communities on a global scale was lacking. Unless the issues of COVID-related mental wellbeing were addressed in a timely manner, such impacts could potentially translate into a range of long-term illnesses with severe economic impacts. As COVID-19 continued to peak in many countries, it was imperative that ongoing planning with mental health support strategies and early identification of psychological distress were realised, because people had the ability to normalise stressful situations when they had access to support networks and resources [12]. Therefore, our study aimed to examine the extent of and the factors associated with psychological distress, the level of fear of COVID-19 and coping strategies amongst a diverse range of community people in multi-country settings.

## Materials and methods

### Study design and settings

We conducted a cross-sectional study across 17 countries utilizing web-based online platforms. Participating countries included Australia, Bangladesh, Egypt, China (Hong Kong), Indonesia, Jordan, Kuwait, Libya, Malaysia, Nepal, Oman, Pakistan, Palestine, Saudi Arabia, Syria, Thailand, and the United Arab Emirates (UAE). Those countries were selected based on the existing collaborative relationships with the first author.

### Study population

Adults aged ≥18 years, living in the participating countries, able to respond to an online questionnaire in English/ Arabic/ Thai/ Nepali were eligible. Thus, study participants included general community members, healthcare professionals, patients, university students and staff. Patients were defined as individuals who attended a general practice or an allied healthcare setting (for any medical condition including COVID-19 related illness) in the previous four weeks at the time of data collection. Frontline or essential service workers were defined as individuals who self-identified themselves as being in contact with patients/clients during the pandemic period.

### Sampling

Sample size was calculated using OpenEpi. Study population and estimated prevalence of stress varied across the participating countries. Therefore, keeping the population size as 100,000,000, assuming 50% prevalence of stress globally, 95% confidence intervals and 80% power, the estimated minimum sample size was 385. That number was the highest possible number, even if the population size and the prevalence of stress varied across countries. Therefore, careful consideration and taking into account the opinion of the cooperating countries, we agreed a minimum sample size of 385 participants for each collaborating country.

### Data collection

An online link was created with a structured survey questionnaire using the Google form. Data were collected in Jun-2020 in Australia, Aug-Sep-2020 in Bangladesh and Malaysia, and during Nov-2020 to Jan-2021 for the other 14 countries. A separate link was created for each language (English, Arabic, Thai and Nepali). The plain language information statement (PLIS) and the consent form appeared on the first screen. Only participants, who provided consent and met the eligibility criteria, could move to the next screen. The subsequent seven screens contained the full study questionnaire, comprising of 39 questions. All responses were anonymous.

The English version of the PLIS, consent form and the study questionnaire were translated into other languages as mentioned above, back-translated to English, reviewed and pilot-tested by the team of local lead investigators for Arabic (Egypt, Saudi Arabia, UAE), Thai (Thailand) and Nepali (Nepal) versions. An invitation with the online survey link and QR code were shared using different social media platforms, online community networks, staff and student email databases of participating universities/hospitals. Text messages using SMS, Viber, WhatsApp were also shared. Flyers containing the QR codes of the study were also distributed and posted in university/healthcare settings. The survey was open to minimise selection bias, so anyone having the survey link could participate in the study; and no incentives were provided for participation in the study.

### Study tool

The structured survey questionnaire was adapted from the previous study conducted in Australia [9]. The survey questionnaire was pre-tested across different electronic devices. Psychological distress was measured using the Kessler Psychological Distress Scale (K-10) having 10-items, [13] fear was measured using the Fear of COVID-19 Scale (FCV-19S) having 7-items, [14] and coping was measured using Brief Resilient Coping Scale (BRCS) having 4-items [15]. Reliability of those tools in the English version was examined in the Australian study, and it was found that they worked for migrants and non-migrants [16].

### Data analyses

The database was downloaded from the Google platform and Stata statistical software Stata/SE V.15.0 for Windows (StataCorp, College Station, USA, 2017) was used for data analyses. Descriptive statistics, including frequencies and percentages, were generated for categorical variables; means and standard deviations (SD) were generated for continuous variables. Psychological distress (based on the K-10 scoring) was categorised into low (score 10-15) and moderate to very high (score 16-50), fear of COVID-19 (based on the FCV-19S scoring) was categorised into low (score 7-21) and high (score 22-35), and coping (based on the BRCS scoring) was categorised into low (score 4-13) and medium to high (score 14-20).

Univariate and multivariate logistic regression analyses were conducted to examine the association between variables. Multivariate analyses were conducted to control potential confounders and the results are presented with odds ratios (ORs), adjusted ORs (AOR) and 95% confidence intervals (CIs). We also tested the sensitivity of analyses by excluding the non-significant association from the univariate model, but no changes were observed in the adjusted model. We investigated potential effect modification between age groups, gender and psychological distress, fear of COVID-19 and coping strategies. The additive log risk model was compared with multiplicative odds ratio model using the likelihood ratio test and Bayesian information criterion. A cut-off of p<0.05 was considered as statistically significant. For the country-wise comparison, we selected the reference country based on the lowest prevalence of moderate to very high psychological distress, lowest prevalence of high level of fear of COVID-19 and lowest prevalence of medium to high resilience coping, then we organised other countries chronologically for each outcome based on the scores prior to conducting the multivariate analyses.

### Ethics

Ethics approval was obtained from the Human Research Ethics Committee from each participating country. The survey was voluntary in nature and participants got the opportunity to have informed decision to participate in the study. Privacy and confidentiality of the collected data were maintained.

## Results

A total of 8987 individuals from 17 countries met the eligibility criteria and consented to participate in the study. However, 8559 of them (95%) completed the questionnaire and were included for analyses. Most countries contributed 6-7% of the study population except Bangladesh (11%) and Saudi Arabia (9%). Mean age (±SD) of the participants was 33 (±13) years and two-thirds (64%) were females. More than one-third (42%) had a source of income during the pandemic, while 51% had their jobs adversely affected by COVID-19. More than one-third (40%) self-identified as frontline or essential service workers, which included 14% doctors and 16% nurses. Only 4% reported having a history of psychiatric or mental health issues. The majority (81%) had never been smokers, and only 11 % reported drinking alcohol in the last four weeks prior to data collection. One in five participants (n=1780; 21%) had direct contact and 952 (11%) participants had indirect contact with known/suspected COVID-19 cases. About 6% tested positive for COVID-19, and 14% reported self-isolating before receiving negative test results. A third of the study participants (n=2752; 33%) visited a healthcare provider (and were defined as ‘patients’ in this study) and one in ten study participants (n=1081; 13%) used healthcare service due to COVID-19 related stress in the last six months. Table 1 shows the characteristics of the study population.

**Table 1:** Characteristics of the study population

Characteristics	Total, n(%)
Total study participants	8559
**Age (in years)**	**7665**
Mean (±SD)	33.3 (12.5)
IQR (25th percentile to 75th percentile)	23-41
**Age groups**	**7664**
18-29 years	3683 (48.1)
30-59 years	3646 (47.6)
≥60 years	335 (4.4)
**Gender**	**8475**
Male	3016 (35.6)
Female	5459 (64.4)
**Country of residence**	**8559**
Australia	587 (6.9)
Bangladesh	962 (11.2)
Egypt	416 (4.9)
Hong Kong	555 (6.5)
Indonesia	541 (6.3)
Jordan	538 (6.3)
Kuwait	417 (4.9)
Libya	114 (1.3)
Malaysia	720 (8.4)
Nepal	311 (3.6)
Oman	437 (5.1)
Pakistan	418 (4.9)
Palestine	417 (4.9)
Saudi Arabia	803 (9.4)
Syria	408 (4.8)
Thailand	498 (5.8)
UAE	417 (4.9)
**Born in the same country of residence**	**8463**
No	1310 (15.3)
Yes	7153 (83.6)
**Living status**	**8441**
Live without family members	1908 (22.6)
Live with family members	6533 (77.4)
**Highest educational/vocational qualification**	**8449**
Primary/Grade 1 to 6	62 (0.7)
Secondary/Higher Secondary/Grade 7 to 12	1546 (18.3)
Certificate/Diploma/Trade qualifications	877 (10.4)
Bachelor/Masters/PhD	5964 (70.6)
**Current employment condition**	**8206**
Unemployed/Housewife/Home maker/Home duties (No source of income)	643 (7.8)
Jobs affected by COVID-19 (lost job/working hours reduced/afraid of job loss)	4148 (50.5)
Have an income source (employed/Government benefits)	3415 (41.6)
**Perceived distress due to change of employment status**	**7268**
A little to none	4712 (61.8)
Moderate to a great deal	2916 (38.2)
**Improved working situation due to change of employment situation**	**5822**
A little to none	4473 (76.8)
Moderate to a great deal	1349 (23.2)
**Self-identification as a frontline or essential service worker**	**8476**
No	5046 (59.5)
Yes	3430 (40.1)
**Self-identification as a healthcare worker**	**6290**
No	3843 (61.1)
Yes, doctor	887 (14.1)
Yes, nurse	1032 (16.4)
Yes, other healthcare worker	528 (8.4)
**COVID-19 impacted financial situation**	**8507**
No impact	3783 (44.5)
Yes, impacted positively	1017 (12.0)
Yes, impacted negatively	3707 (43.6)
**Affected by the change in financial situation**	**6122**
Not at all	1397 (22.8)
Unsure at this time	912 (14.9)
Somewhat	2770 (45.2)
A great extent	1043 (17.0)
**Co-morbidities**	**8416**
No	5975 (71.0)
Mental health issue	362 (4.3)
Other co-morbidity	2079 (24.7)
**Co-morbidities**	**8416**
No	5975 (71.0)
Single co-morbidity	1547 (19.3)
Multiple co-morbidities	474 (5.9)
**Smoking**	**8507**
Never smoker	6910 (81.2)
Ever smoker (Daily/Non-daily/Ex)	1597 (18.8)
**Increased smoking over the last 6 months**	**1018**
No	535 (52.6)
Yes	483 (47.4)
**Current alcohol drinking (last 4 weeks)**	**8365**
No	7435 (88.9)
Yes	930 (11.1)
**Increased alcohol drinking over the last 6 months**	**921**
No	645 (70.0)
Yes	276 (30.0)
**Contact with known/suspected case of COVID-19**	**8341**
No	4899 (58.7)
Unsure	710 (8.5)
Yes, indirect contact	952 (11.4)
Yes, provided direct care	1780 (21.3)
**Experience related to COVID-19 pandemic (multiple responses possible)**	**8171**
No known exposure to COVID-19	6337 (77.6)
Tested positive for COVID-19	494 (6.0)
Tested negative for COVID-19 by self-isolated	1135 (13.9)
Had recent overseas travel history and was in quarantine	205 (2.5)
**Self-identification as a patient (visited a healthcare provider in the last 6 months)**	**8322**
No	5570 (66.9)
Yes	2752 (33.1)
**Healthcare service use in the last 6 months**	**2727**
In-person visit to a healthcare provider	1896 (69.5)
Telehealth consultation/Use of national helpline	636 (23.3)
Used both services	195 (7.2)
**Perceived mental health status**	**6290**
Poor to fair	1753 (27.9)
Good to excellent	4537 (72.1)
**Healthcare service use to overcome COVID-19 related stress in the last 6 months**	**8264**
No	7183 (86.9)
Yes	1081 (13.1)
**Type of healthcare service used to overcome COVID-19 related stress in the last 6 months**	**1041**
Consulted a GP	356 (34.2)
Consulted a Psychologist	53 (5.1)
Consulted a Psychiatrist	63 (6.1)
Used specialised mental healthcare settings	26 (2.5)
Used mental health resources	93 (8.9)
Used mental health resources available through media	171 (16.4)
Used mental health support services	79 (7.6)
Used combination of services	199 (19.1)

More than two-thirds of the study participants (n=5846; 69%) experienced moderate to very high levels of psychological distress, a quarter (n=2066; 24%) had high levels of fear of COVID-19, and 4815 (57%) exhibited medium to high resilient coping (Tables S.1, S.2, S.3).

### Psychological distress

The univariate analyses showed reasonable evidence against the null hypothesis of no association between moderate to very high levels of psychological distress and a number of variables (Table 2). However, when adjusted for potential confounders, being female, perceived distress due to change of employment status, self-identification as a doctor, being affected by the change of financial situation, comorbidity with mental health conditions, unsure and indirect contact with COVID-19 patient, being a patient, use of healthcare service to overcome COVID-related stress, and higher levels of fear of COVID-19 were found to be associated with moderate to very high levels of psychological distress. We did not identify any effect modification between age groups, gender, and psychological distress.

**Table 2:** Predictors for psychological distress among the study participants (based on the K-10 score)

Characteristics	Low (score 10-15)		Moderate to Very High (score 16-50)		Unadjusted analyses			Adjusted analyses		
	n	%	n	%	p	ORs	95% CIs	p	AORs	95% CIs
**Age groups**	**2434**	**32.1**	**5157**	**67.9**						
18-29 years	775	21.1	2884	78.8	Ref			Ref		
30-59	1429	39.7	2170	60.3	**<0.001**	**0.41**	**0.37-0.45**	**<0.001**	**0.50**	**0.41-0.61**
≥60 years	230	69.1	103	30.9	**<0.001**	**0.12**	**0.08-0.15**	**<0.001**	**0.15**	**0.09-0.23**
**Gender**	**2622**	**31.1**	**5810**	**68.9**						
Male	1100	36.7	1898	63.3	Ref			Ref		
Female	1522	28	3912	71.9	**<0.001**	**1.50**	**1.36-1.64**	**0.003**	**1.31**	**1.09-1.57**
**Born in the same country of residence**	**2611**	**31**	**5807**	**68.9**						
No	421	32.7	864	67.2	Ref			Ref		
Yes	2190	30.7	4943	69.3	0.118	1.06	0.96-1.18	0.193	1.18	0.92-1.52
**Living status**	**2609**	**31.1**	**5790**	**68.9**						
Live without family members	608	32.1	1289	67.9	Ref			Ref		
Live with family members	2001	30.9	4501	69.2	0.133	1.09	0.97-1.24	0.064	1.25	0.99-1.56
**Highest educational/vocational qualification**	**2603**	**30.9**	**5803**	**69.03**						
Primary/Grade 1 to 6	20	33.9	39	66.1	Ref			Ref		
Secondary/Higher Secondary/Grade 7 to 12	373	24.2	1168	75.8	0.100	1.61	0.91-2.83	0.375	0.53	0.13-2.14
Certificate/Diploma/Trade qualifications	269	30.9	601	68.1	0.605	1.16	0.65-2.06	0.231	0.43	0.11-1.72
Bachelor/Masters/PhD	1941	32.7	3995	67.3	0.848	1.06	0.61-1.84	0.247	0.44	0.11-1.75
**Current employment condition**	**2565**	**31.4**	**5597**	**68.5**						
Unemployed/Housewife/Home maker/Home duties (No source of income)	242	37.6	401	**62.4**	Ref			Ref		
Jobs affected by COVID-19 (lost job/working hours reduced/afraid of job loss)	1499	36.4	2623	63.6	0.481	1.06	0.89-1.26	No estimates due to small number		
Have an income source (employed/Government benefits)	824	24.3	2573	75.4	**<0.001**	**1.88**	**1.58-2.25**	**0.003**	**1.35**	**1.10-1.63**
**Perceived distress due to change of employment status**	**2317**	**30.5**	**5300**	**69.6**						
A little to none	1735	36.8	2970	63.1	Ref			Ref		
Moderate to a great deal	582	19.9	2330	80.01	**<0.001**	**2.38**	**2.1-2.61**	**<0.001**	**1.56**	**1.29-1.90**
**Improved working situation due to change of employment status**	1730	**29.7**	**4092**	**70.3**						
A little to none	1373	30.6	3100	69.3	Ref			Ref		
Moderate to a great deal	357	26.5	992	73.5	**0.022**	**1.23**	**1.07-1.41**	0.723	0.97	0.80-1.18
**Self-identification as a frontline or essential service worker**	**2621**	**31.1**	**5823**	**68.9**						
No	1588	31.6	3437	68.4	Ref			Ref		
Yes	1033	30.2	2386	69.7	0.084	1.07	0.98-1.19	0.830	0.98	0.79-1.21
**Self-identification as a healthcare worker**	**1874**	**29.8**	**4416**	**70.2**						
No	1072	27.8	2771	72.1	Ref			Ref		
Yes, doctor	261	29.4	626	70.6	0.291	0.92	0.78-1.08	**0.028**	**1.43**	**1.04-1.97**
Yes, nurse	395	38.3	637	61.7	**<0.001**	**0.63**	**0.54-0.72**	0.375	1.13	0.86-1.5
Yes, other healthcare worker	146	27.6	382	72.4	0.893	1.01	0.82-1.25	0.521	1.11	0.81-1.52
**COVID-19 impacted financial situation**	**2634**	**31.1**	**5845**	**68.9**						
No impact	1479	39.2	2297	60.8	Ref			Ref		
Yes, impacted positively	292	28.7	725	71.3	**<0.001**	**1.59**	**1.37-1.86**	0.330	1.14	0.88-1.48
Yes, impacted negatively	863	23.4	2823	76.6	**<0.001**	**2.10**	**1.89-2.32**	0.770	1.03	0.84-1.27
**Affected by the change in financial situation**	**1814**	**29.6**	**4308**	**70.4**						
Not at all	690	49.4	707	50.6	Ref			Ref		
Unsure	268	29.4	644	70.6	**<0.001**	**2.35**	**1.96-2.80**	**<0.001**	**1.69**	**1.32-2.16**
Somewhat	710	25.6	2060	74.4	**<0.001**	**2.83**	**2.47-3.24**	**<0.001**	**1.64**	**1.32-2.03**
A great extent	146	14	897	86	**<0.001**	**5.99**	**4.89-7.35**	**<0.001**	**2.36**	**1.72-3.23**
**Co-morbidities**	**2601**	**31.1**	**5770**	**68.9**						
No	1926	32.3	4020	67.6	Ref			Ref		
Psychiatric/Mental health problem	31	8.7	327	91.3	**<0.001**	**5.04**	**3.47-7.32**	**0.019**	**3.02**	**1.20-7.60**
Other co-morbidities*	644	31.2	1423	68.8	0.436	1.04	0.94-1.17	0.147	1.30	0.91-1.82
**Co-morbidities**	**2465**	**30.9**	**5502**	**69.1**						
No	1926	32.4	4020	67.6	Ref			Ref		
Single co-morbidity	411	26.6	1136	73.4	**0.001**	**1.32**	**1.17-1.50**	0.859	0.97	0.67-1.40
Multiple co-morbidities	128	27	346	73	0.114	1.30	1.05-1.60	No estimates due to small number		
**Perceived status of own mental health**	**1874**	**29.8**	**4416**	**70.2**						
Poor to Fair	131	7.5	1622	92.5	Ref			Ref		
Good to Excellent	1743	38.4	2794	61.6	**<0.001**	**0.13**	**0.11-0.16**	**<0.001**	**0.17**	**0.13-0.22**
**Smoking**	**2634**	**31.1**	**5846**	**68.9**						
Never smoker	2226	32.3	4668	67.6	Ref			Ref		
Ever smoker (Daily/Non-daily/Ex)	408	25.7	1178	74.3	**<0.001**	**1.38**	**1.22-1.56**	0.434	1.10	0.87-1.39
**Increased smoking over the last 6 months**	**206**	**20.3**	**808**	**79.7**						
No	151	28.2	384	71.9	Ref			Not included in multivariate model		
Yes	55	11.5	424	88.5	**<0.000**	**3.03**	**2.16-4.25**			
**Current alcohol drinking (last 4 weeks)**	**2583**	**30.9**	**5755**	**69.02**						
No	2314	31.2	5104	68.7	Ref			Ref		
Yes	269	29.2	651	70.7	0.199	1.10	0.95-1.28	0.069	1.29	0.99-1.68
**Increased alcohol drinking over the last 6 months**	**266**	**29.2**	**645**	**70.8**						
No	235	36.9	404	63.2	Ref			Not included in multivariate model		
Yes	31	11.4	241	88.6	**<0.001**	**4.52**	**3.01-6.80**			
**Contact with known/suspected case of COVID-19**	**2574**	**30.9**	**5743**	**69.1**						
No	1754	35.9	3127	64.1	Ref			Ref		
Unsure	141	19.9	567	80.1	**<0.001**	**2.26**	**1.85-2.73**	**<0.001**	**1.80**	**1.36-2.40**
Yes, had indirect contact	223	23.4	729	76.5	**<0.001**	**1.83**	**1.55-2.16**	**0.019**	**1.32**	**1.04-1.67**
Yes, provided direct care	456	25.6	1320	74.3	**<0.001**	**1.63**	**1.44-1.85**	0.814	1.03	0.81-1.30
**Experience related to COVID-19 pandemic**	**2518**	**30.9**	**5631**	**69.1**						
No known exposure to COVID-19	2095	33.2	4224	66.8	Ref			Ref		
Tested positive for COVID-19	124	25.2	369	74.8	**<0.001**	**1.48**	**1.2-1.82**	0.988	1.00	0.72-1.38
Tested negative for COVID-19 by self-isolated	256	22.6	876	77.3	**<0.001**	**1.69**	**1.45-1.97**	0.086	1.24	0.97-1.58
Had recent overseas travel history and was in quarantine	43	20.9	162	79.02	**0.002**	**1.87**	**1.32-2.62**	0.696	1.12	0.64-1.93
**Self-identification as a patient (visited a healthcare provider in the last 6 months)**	**2579**	**31.1**	**5719**	**68.9**						
No	1945	35.1	3606	64.9	Ref			Ref		
Yes	634	23.1	2113	76.9	**<0.001**	**1.80**	**1.61-2.00**	**<0.001**	**1.67**	**1.40-1.99**
**Healthcare service use in the last 6 months**	**646**	**23.7**	**2079**	**76.3**						
In-person visit to a healthcare provider	493	26.1	1401	73.9	Ref			Ref		
Telehealth consultation/Use of national helpline	120	18.9	516	81.1	**<0.001**	**1.51**	**1.21-1.89**	Not included in multivariate model		
Used both services	33	16.9	162	83.1	**0.005**	**1.72**	**1.17-2.54**			
**Level of fear of COVID-19 (FCV-19S categories)**	**2634**	**31.1**	**5845**	**68.9**						
Low (score 7-21)	2328	36.3	4088	63.7	Ref			Ref		
High (score 22-35)	306	14.8	1757	85.2	**<0.001**	**3.27**	**2.87-3.73**	**<0.001**	**3.26**	**2.57-4.13**
**Level of coping (BRCS categories)**	**2633**	**31.1**	**5840**	**68.9**						
Low resilient copers (score 4-13)	1011	27.6	2648	72.4	Ref			Ref		
Medium to high resilient copers (score 14-20)	1622	33.7	3192	66.3	**<0.001**	**0.75**	**0.69-0.82**	0.637	0.96	0.81-1.14
**Healthcare service use to overcome COVID-19 related stress in the last 6 months**	**2560**	**31**	**5697**	**69**						
No	2422	33.7	4754	66.3	Ref			Ref		
Yes	138	12.8	943	69	**<0.001**	**3.48**	**2.89-4.19**	**<0.001**	**1.99**	**1.45-2.72**

*Adjusted for: age, gender, smoking, alcohol intake, living status, place of birth, country, education, employment status, employment stress, healthcare worker, financial impact, contact with COVID-19 case, experience due to COVID-19 and self-identification as a patient*

### Levels of fear

Similar to psychological distress, participants from all 17 countries demonstrated significant levels of fear to COVID 19 (Table 3). After adjusting for potential confounders, high levels of fear were associated with being aged 30-59 years, being female, perceived distress due to a change of employment status, self-identification as a frontline or essential service worker, being affected by the change of financial situation, having comorbidities, drinking alcohol in the previous four weeks, unsure contact with a COVID-19 case, health service use to overcome COVID-related stress, and having moderate to very high levels of psychological distress. We did observe some effect modification with gender and fear of COVID-19 (contact with a COVID-19 patient) (data not shown).

**Table 3:** Predictors for fear of COVID-19 among the study participants (based on the FCV-19S score)

Characteristics	Low (score 7-21)		High (score 22-35)		Unadjusted analyses			Adjusted analyses		
	n	%	n	%	p	ORs	95% CIs	p	AORs	95% CIs
**Age groups**	**5710**	**75.20**	**1886**	**24.8**						
18-29 years	2777	75.8	883	24.1	Ref			Ref		
30-59 years	2661	73.8	942	26.1	**0.047**	**1.11**	**1.00-1.24**	**0.004**	**1.35**	**1.10-1.64**
≥60 years	272	81.6	61	18.3	**0.017**	**0.71**	**0.53-0.94**	0.184	1.40	0.86-2.30
**Gender**	**6383**	**75.6**	**2055**	**24.4**						
Male	2305	76.8	695	23.20	Ref			Ref		
Female	4078	74.9	1360	25.1	0.059	1.11	0.99-1.23	**0.001**	**1.51**	**1.25-1.83**
**Born in the same country of residence**	**6365**	**75.5**	**2059**	**24.4**						
No	933	72.4	355	27.6	Ref			Ref		
Yes	5432	76.1	1704	23.8	**0.005**	**0.82**	**0.72-0.94**	**0.001**	**0.66**	**0.51-0.85**
**Living status**	**6354**	**75.6**	**2050**	**24.4**						
Live without family members	1322	69.6	577	30.4	Ref			Ref		
Live with family members	5032	77.4	1473	22.6	**<0.001**	**0.67**	**0.6-0.75**	0.431	1.10	0.86-1.41
**Highest educational/vocational qualification**	**6359**	**75.6**	**2052**	**24.4**						
Primary/Grade 1 to 6	47	79.6	12	20.3	Ref			Ref		
Secondary/Higher Secondary/Grade 7 to 12	1176	76.3	366	23.7	0.547	1.22	0.64-2.32	0.569	1.41	0.44-4.55
Certificate/Diploma/Trade qualifications	626	71.8	245	28.1	0.198	1.53	0.8-2.93	0.298	1.87	0.57-6.09
Bachelor/Masters/PhD	4510	75.9	1429	24.1	0.506	1.24	0.66-2.35	0.689	1.27	0.40-4.05
**Current employment condition**	**6174**	**75.6**	**1994**	**24.4**						
Unemployed/Housewife/Home maker/Home duties (No source of income)	433	67.3	210	32.6	Ref			Ref		
Jobs affected by COVID-19 (lost job/working hours reduced/afraid of job loss)	3304	80.1	821	19.9	**<0.001**	**0.51**	**0.42-0.61**	No estimate due to small number		
Have an income source (employed/Government benefits)	2437	71.7	963	28.3	**0.026**	**0.81**	**0.68-0.98**	0.588	1.05	0.87-1.27
**Perceived distress due to change of employment status**	**5772**	**75.7**	**1847**	**24.2**						
A little to none	3767	80.1	939	19.9	Ref			Ref		
Moderate to a great deal	2005	68.8	908	31.2	**<0.001**	**1.82**	**1.63-2.02**	**<0.001**	**1.52**	**1.27-1.82**
**Improved working situation due to change of employment status**	4570	78.5	1251	21.5						
A little to none	3566	79.7	906	20.3	Ref			Ref		
Moderate to a great deal	1004	74.4	345	25.6	**<0.001**	**1.35**	**1.17-1.56**	0.401	1.08	0.9-1.32
**Self-identification as a frontline or essential service worker**	**6398**	**75.7**	**2052**	**24.3**						
No	3839	76.3	1191	23.7	Ref			Ref		
Yes	2559	74.8	861	25.2	0.115	1.08	0.99-1.2	**0.001**	**1.47**	**1.20-1.82**
**Self-identification as a healthcare worker**	**4950**	**78.7**	**1339**	**21.3**						
No	2990	77.8	853	22.2	Ref			Ref		
Yes, doctor	712	80.4	174	19.6	0.096	0.86	0.71-1.03	**<0.001**	**0.55**	**0.41-0.76**
Yes, nurse	838	81.2	194	18.8	**0.018**	**0.81**	**0.68-0.97**	0.053	0.75	0.56-1.01
Yes, other healthcare worker	410	77.6	118	22.4	0.937	1.01	0.81-1.26	0.131	0.79	0.58-1.07
**COVID-19 impacted financial situation**	**6418**	**75.6**	**2066**	**24.4**						
No impact	3053	80.8	725	19.2	Ref			Ref		
Yes, impacted positively	768	75.5	249	24.5	**<0.001**	**1.37**	**1.16-1.61**	0.075	1.29	0.98-1.70
Yes, impacted negatively	2597	70.4	1092	29.6	**<0.001**	**1.77**	**1.6-1.97**	**0.004**	**1.36**	**1.11-1.68**
**Affected by the change in financial situation**	4813	78.6	1308	21.4						
Not at all	1201	85.9	196	14	Ref			Ref		
Unsure	724	79.4	188	20.6	**<0.001**	**1.59**	**1.28-1.98**	0.149	1.23	0.93-1.64
Somewhat	2169	78.3	600	21.7	**<0.001**	**1.69**	**1.42-2.02**	**0.033**	**1.32**	**1.02-1.08**
A great extent	719	68.9	324	31.1	**<0.001**	**2.76**	**2.26-3.37**	**0.021**	**1.44**	**1.06-1.96**
**Co-morbidities**	**6345**	**75.7**	**2032**	**24.3**						
No	4645	78.1	1303	21.9	Ref			Ref		
Psychiatric/Mental health problem	248	68.7	113	31.3	**<0.001**	**1.62**	**1.29-2.05**	0.984	1.00	0.64-1.60
Other co-morbidities*	1452	70.2	616	29.8	**<0.001**	**1.51**	**1.35-1.7**	**0.001**	**1.71**	**1.25-2.32**
**Co-morbidities**	6059	76.1	1910	23.9						
No	4645	78.1	1303	21.9	Ref			Ref		
Single co-morbidity	1096	70.9	451	29.2	**<0.001**	**1.47**	**1.29-1.66**	**0.021**	**0.69**	**0.51-0.95**
Multiple co-morbidities	318	67.1	156	32.9	**<0.001**	**1.75**	**1.43-2.14**	No estimate due to small number		
**Perceived status of own mental health**	4950	78.7	1339	21.3						
Poor to Fair	1190	67.9	563	32.1	Ref			Ref		
Good to Excellent	3760	82.9	776	17.1	**<0.001**	**0.44**	**0.39-0.5**	**<0.001**	**0.72**	**0.60-0.86**
**Smoking**	**6420**	**75.6**	**2065**	**24.3**						
Never smoker	5251	76.1	1647	23.8	Ref			Ref		
Ever smoker (Daily/Non-daily/Ex)	1169	73.6	418	26.3	**0.039**	**1.14**	**1.01-1.30**	0.708	1.04	0.84-1.31
**Increased smoking over the last 6 months**	**758**	**74.7**	**256**	**25.3**						
No	418	78.1	117	21.9	Ref			Not included in multivariate model		
Yes	340	70.9	139	29	**0.009**	**1.46**	**1.1-1.94**			
**Current alcohol drinking (last 4 weeks)**	**6309**	**75.6**	**2035**	**24.4**						
No	5646	76.1	1776	23.9	Ref			Ref		
Yes	663	71.9	259	28.1	**0.006**	**1.24**	**1.07-1.45**	**0.038**	**1.33**	**1.02-1.73**
**Increased alcohol drinking over the last 6 months**	**658**	**72.1**	**255**	**27.9**						
No	511	79.7	130	20.3	Ref			Not included in multivariate model		
Yes	147	54.1	125	45.9	**<0.001**	**3.34**	**2.46-4.54**			
**Contact with known/suspected case of COVID-19**	**6292**	**75.6**	**2031**	**24.4**						
No	3769	771	1117	22.9	Ref			Ref		
Unsure	488	68.8	221	31.2	**<0.001**	**1.53**	**1.29-1.82**	**0.006**	**1.41**	**1.10-1.80**
Yes, had indirect contact	722	75.8	230	24.2	0.384	1.07	0.92-1.26	0.713	1.04	0.86-1.35
Yes, provided direct care	1313	73.9	463	26.1	0.007	1.19	1.04-1.35	0.782	0.97	0.76-1.23
**Experience related to COVID-19 pandemic**	**6155**	**75.5**	**2000**	**24.5**						
No known exposure to COVID-19	4833	76.4	1490	23.6	Ref			Ref		
Tested positive for COVID-19	391	79.2	103	20.8	0.170	0.85	0.68-1.07	0.175	0.80	0.57-1.11
Tested negative for COVID-19 by self-isolated	791	69.8	342	30.2	**<0.001**	**1.40**	**1.22-1.61**	0.336	1.12	0.89-1.41
Had recent overseas travel history and was in quarantine	140	68.3	65	31.7	**0.007**	**1.51**	**1.12-2.03**	0.808	0.93	0.54-1.61
**Self-identification as a patient (visited a healthcare provider in the last 6 months)**	**6273**	**75.5**	**2031**	**24.5**						
No	4247	76.5	1308	23.6	Ref			Ref		
Yes	2026	73.7	723	26.3	**0.006**	**1.16**	**1.04-1.29**	0.217	0.90	0.76-1.06
**Healthcare service use in the last 6 months**	**1973**	**72.4**	**754**	**27.6**						
In-person visit to a healthcare provider	1413	74.5	483	25.5	Ref			Ref		
Telehealth consultation/Use of national helpline	426	66.9	210	33	**<0.001**	**1.44**	**1.19-1.75**	Not included in multivariate model		
Used both services	134	68.7	61	31.3	0.079	1.33	0.97-1.83			
**Level of psychological distress (K10 categories)**	**6416**	**75.7**	**2063**	**24.3**						
Low (score 10-15)	2328	88.4	306	11.6	Ref			Ref		
Moderate to Very High (score 16-50)	4088	69.9	1757	30.1	**<0.001**	**3.26**	**2.87-3.72**	**<0.001**	**3.36**	**2.67-4.23**
**Level of coping (BRCS categories)**	**6418**	**75.7**	**2061**	**24.3**						
Low resilient copers (score 4-13)	2647	72.2	1018	27.8	Ref			Ref		
Medium to high resilient copers (score 14-20)	3771	78.3	1043	21.7	**<0.001**	**0.72**	**0.65-0.80**	**<0.001**	**0.74**	**0.63-0.87**
**Healthcare service use to overcome COVID-19 related stress in the last 6 months**	**6243**	**75.6**	**2020**	**24.5**						
No	5595	77.9	1587	22.1	Ref			Ref		
Yes	648	59.9	433	40.1	**<0.001**	**2.35**	**2.06-2.70**	**<0.001**	**2.42**	**1.96-3.01**

*Adjusted for: age, gender, smoking, alcohol intake, living status, place of birth, country, education, employment status, employment stress, healthcare worker, financial impact, contact with COVID-19 case, experience due to COVID-19 and self-identification as a patient*

### Coping strategies

Table 4 shows the univariate analyses identifying significant association between medium to high resilient coping and other variables. From the multivariate analyses, we identified that participants who were ≥60 years old, self-identification as a nurse, whose financial situation was impacted negatively, who perceived their own mental health as good to excellent, who had indirect contact and direct contact with known or suspected cases of COVID-19, and who visited a healthcare provider in the previous six months were more likely to have medium to high resilient coping. We did not identify any effect modification between age group, gender, and coping strategies (data not shown).

**Table 4:** Predictors for coping among the study participants (based on the BRCS score)

Characteristics	Low (score 4-13)		Medium to High (score 14-20)		Unadjusted analyses			Adjusted analyses		
	n	%	n	%	p	ORs	95% CIs	p	AORs	95% CIs
**Age groups**	**3247**	**42.8**	**4344**	**57.2**						
18-29 years	1581	43.3	2074	56.7	Ref			Ref		
30-59 years	1543	42.8	2060	57.2	0.711	1.02	0.93-1.12	0.329	1.08	0.92-1.28
≥60 years	123	36.9	210	63.1	**0.026**	**1.30**	**1.03-1.64**	**0.011**	**1.66**	**1.12-2.44**
**Gender**	**3640**	**43.2**	**4792**	**56.8**						
Male	1323	44.1	1675	55.9	Ref			Ref		
Female	2317	42.6	3117	57.4	0.186	1.07	0.97-1.17	0.235	0.91	0.79-1.06
**Born in the same country of residence**	**3635**	**43.2**	**4783**	**56.8**						
No	649	50.4	639	49.6	Ref			Ref		
Yes	2986	41.8	4144	58.1	**<0.001**	**1.41**	**1.25-1.59**	0.124	0.85	0.69-1.05
**Living status**	3614	43	4784	56.9						
Live without family members	812	42.7	1087	57.2	Ref			Ref		
Live with family members	2802	43.1	3697	56.9	0.780	0.99	0.89-1.1	0.106	0.85	0.7-1.04
**Highest educational/vocational qualification**	**3622**	**43.1**	**4783**	**56.9**						
Primary/Grade 1 to 6	30	50.8	29	49.2	Ref			Ref		
Secondary/Higher Secondary/Grade 7 to 12	673	43.7	868	56.3	0.277	1.33	0.8-2.24	0.537	1.35	0.52-3.48
Certificate/Diploma/Trade qualifications	409	47.2	458	57.7	0.585	1.16	0.69-1.96	0.871	1.08	0.42-2.81
Bachelor/Masters/PhD	2510	42.3	3428	57.7	0.187	1.41	0.85-2.36	0.583	1.30	0.51-3.32
**Current employment condition**	**3523**	**43.2**	**4639**	**56.8**						
Unemployed/Housewife/Home maker/Home duties (No source of income)	260	40.4	383	59.5	Ref			Ref		
Jobs affected by COVID-19 (lost job/working hours reduced/afraid of job loss)	1734	42.1	2391	57.9	0.444	0.94	0.797-1.11	No estimate due to small number		
Have an income source (employed/Government benefits)	1529	45.1	1865	54.9	**0.031**	**0.84**	**0.69-0.99**	0.354	0.93	0.8-1.09
**Perceived distress due to change of employment status**	**3095**	**40.6**	**4522**	**59.4**						
A little to none	1815	38.6	2889	61.4	Ref			Ref		
Moderate to a great deal	1280	43.9	1633	56.1	**<0.001**	**0.80**	**0.73-0.88**	**0.030**	**0.82**	**0.68-0.98**
**Improved working situation due to change of employment status**	2291	39.4	3528	60.6						
A little to none	1753	39.2	2717	60.8	Ref			Ref		
Moderate to a great deal	538	39.8	811	60.1	0.662	0.98	0.86-1.1	0.342	1.09	0.92-1.28
**Self-identification as a frontline or essential service worker**	**3646**	**43.2**	**4798**	**56.8**						
No	2155	42.9	2869	57.1	Ref			Ref		
Yes	1491	43.6	1929	56.4	0.522	0.97	0.87-1.06	0.525	0.94	0.8-1.13
**Self-identification as a healthcare worker**	2482	39.5	3801	60.5						
No	1578	41.1	2259	58.9	Ref			Ref		
Yes, doctor	331	37.4	555	62.6	**0.040**	**1.17**	**1.01-1.36**	0.417	0.90	0.70-1.16
Yes, nurse	371	35.9	661	64.1	**0.003**	**1.24**	**1.08-1.44**	**0.029**	**1.30**	**1.03-1.65**
Yes, other healthcare worker	202	38.3	326	61.7	0.209	1.13	0.94-1.36	0.280	1.15	0.90-1.48
**COVID-19 impacted financial situation**	**3663**	**43.2**	**4815**	**56.8**						
No impact	**1613**	**42.8**	**2160**	**57.3**	Ref			Ref		
Yes, impacted positively	**413**	**40.7**	**603**	**59.4**	0.229	1.10	0.95-1.26	0.851	0.98	0.80-1.23
Yes, impacted negatively	1637	44.4	2052	55.6	0.157	0.94	0.85-1.03	**<0.001**	**1.37**	**1.16-1.62**
**Affected by the change in financial situation**	2403	39.3	3712	60.7						
Not at all	523	37.4	874	62.6	Ref			Ref		
Unsure	385	42.4	523	57.6	**0.017**	**0.81**	**0.69-0.96**	**0.004**	**0.74**	**0.60-0.90**
Somewhat	1051	37.9	1716	62	0.732	0.98	0.86-1.12	0.398	0.92	0.78-1.14
A great extent	444	42.6	599	57.4	**0.010**	**0.81**	**0.69-0.95**	0.151	0.83	0.66-1.07
**Co-morbidities**	**3630**	**43.4**	**4741**	**56.6**						
No	2458	41.4	3488	58.7	Ref			Ref		
Psychiatric/Mental health problem	223	62.5	134	37.5	**<0.001**	**0.42**	**0.33-0.52**	0.431	0.85	0.57-1.27
Other co-morbidities*	949	45.9	1119	54.1	**<0.001**	**0.82**	**0.73-0.91**	0.324	1.15	0.88-1.50
**Co-morbidities**	**3321**	**41.7**	**4642**	**58.3**						
No	2458	41.3	3488	58.7	Ref			Ref		
Single co-morbidity	674	43.6	873	56.4	0.113	0.91	0.81-1.02	0.149	0.82	0.62-1.09
Multiple co-morbidities	189	40.2	281	59.8	0.633	1.05	0.87-1.27	No estimate due to small number		
**Perceived status of own mental health**	2482	39.5	3801	60.5						
Poor to Fair	**913**	**52.1**	**839**	**47.8**	Ref			Ref		
Good to Excellent	1569	34.6	2962	65.4	**<0.001**	**2.05**	**1.83-2.3**	**<0.001**	**1.97**	**1.70-2.30**
**Smoking**	**3665**	**43.2**	**4814**	**56.8**						
Never smoker	2912	42.2	3982	57.8	Ref			Ref		
Ever smoker (Daily/Non-daily/Ex)	753	47.5	832	52.5	**<0.001**	**0.81**	**0.72-0.90**	0.533	1.06	0.88-1.28
**Increased smoking over the last 6 months**	**447**	**44.2**	**565**	**55.8**						
No	234	43.7	301	56.3	Ref			Ref		
Yes	213	44.6	264	55.4	0.770	0.96	0.75-1.23	Not included in multivariate model		
**Current alcohol drinking (last 4 weeks)**	**3595**	**43.1**	**4743**	**56.8**						
No	3089	41.6	4328	58.4	Ref			Ref		
Yes	506	54.9	415	45.1	**<0.001**	**0.59**	**0.50-0.66**	0.532	0.93	0.74-1.17
**Increased alcohol drinking over the last 6 months**	**499**	**54.7**	**413**	**45.3**						
No	310	48.4	330	51.7	Ref			Not included in multivariate model		
Yes	189	69.5	83	30.5	**<0.001**	**0.40**	**0.31-0.56**			
**Contact with known/suspected case of COVID-19**	**3578**	**43**	**4739**	**56.9**						
No	2223	45.5	2662	54.5	Ref			Ref		
Unsure	333	46.9	376	53	0.470	0.94	0.81-1.1	0.297	0.90	0.73-1.1
Yes, had indirect contact	353	37.3	594	62.7	**<0.001**	**1.41**	**1.21-1.63**	**0.004**	**1.33**	**1.10-1.62**
Yes, provided direct care	669	37.7	1107	62.3	**<0.001**	**1.37**	**1.22-1.53**	**<0.001**	**1.45**	**1.19-1.77**
**Experience related to COVID-19 pandemic**	3497	42.9	4652	57.1						
No known exposure to COVID-19	2739	43.4	3580	56.6	Ref			Ref		
Tested positive for COVID-19	184	37.3	310	62.7	**0.008**	**1.29**	**1.07-1.56**	0.259	0.86	0.65-1.12
Tested negative for COVID-19 by self-isolated	480	42.4	651	57.6	0.571	1.03	0.91-1.18	**0.012**	**0.78**	**0.64-0.95**
Had recent overseas travel history and was in quarantine	94	45.8	111	54.2	0.476	0.90	0.68-1.2	0.312	0.80	0.51-1.24
**Self-identification as a patient (visited a healthcare provider in the last 6 months)**	**3564**	**42.9**	**4734**	**57.1**						
No	2466	44.4	3089	55.6	Ref			Ref		
Yes	1098	40.1	1645	59.9	**0.001**	**1.20**	**1.09-1.31**	**0.012**	**1.20**	**1.04-1.28**
**Healthcare service use in the last 6 months**	**1089**	**40**	**1633**	**59.9**						
In-person visit to a healthcare provider	730	38.5	1165	61.5	Ref			Ref		
Telehealth consultation/Use of national helpline	277	43.5	359	56.5	**0.025**	**0.82**	**0.67-0.97**	Not included in multivariate model		
Used both services	82	42.9	109	57.1	0.234	0.83	0.62-1.13			
**Level of psychological distress (K10 categories)**	**3659**	**43.2**	**4814**	**56.8**						
Low (score 10-15)	1011	38.4	1622	61.6	Ref			Ref		
Moderate to Very High (score 16-50)	2648	45.4	3192	54.6	**<0.001**	**0.74**	**0.67-0.81**	0.498	0.95	0.81-1.11
**Level of fear of COVID-19 (FCV-19S categories)**	**3665**	**43.2**	**4814**	**56.7**						
Low (score 7-21)	2647	41.2	3771	58.8	Ref			Ref		
High (score 22-35)	1018	49.4	1043	50.6	**<0.001**	**0.71**	**0.64-0.78**	**<0.001**	**0.72**	**0.61-0.85**
**Healthcare service use to overcome COVID-19 related stress in the last 6 months**	**3546**	**42.9**	**4718**	**57.1**						
No	3049	42.4	4134	57.6	Ref			Ref		
Yes	497	45.9	584	54	**0.030**	**0.87**	**0.76-0.99**	0.375	0.91	0.75-1.12

*Adjusted for: age, gender, smoking, alcohol intake, living status, place of birth, country, education, employment status, employment stress, healthcare worker, financial impact, contact with COVID-19 case, experience due to COVID-19 and self-identification as a patient*

### Country-wise findings

Country-wise analyses (Table 5) showed that moderate to very high levels of psychological distress was common in all 17 countries. The lowest prevalence (46%) was reported from Thailand and the highest (91%) from Egypt. When other countries were compared considering Thailand as the baseline, it was found that participants from 10 countries (Hong Kong, Oman, Libya, Kuwait, Saudi Arabia, UAE, Jordan, Syria, Palestine and Egypt), demonstrated statistically significant high psychological distress. Prevalence on high levels of fear of COVID-19 varied across 17 countries (Libya: 9%, Bangladesh: 38%). Participants from four countries (Oman, Indonesia, Hong Kong and Pakistan) exhibited higher levels of fear of COVID-19 compared to the participants from Libya. Finally, participants from 12 countries (Jordan, Egypt, Saudi Arabia, Kuwait, Hong Kong, UAE, Palestine, Thailand, Oman, Nepal, Indonesia and Syria) demonstrated statistically significant medium to high resilience coping compared to those from Australia.

**Table 5:** Country-wise analyses for high psychological distress, fear of COVID-19 and coping among the study participants

**Characteristics**	**K-10 Score**				**Unadjusted analyses**			**Adjusted analyses**		
	**Low (score 10-15)**		**Moderate to Very High (score 16-50)**							
	**n**	**%**	**n**	**%**	**p**	**ORs**	**95% CIs**	**p**	**AORs**	**95% CIs**
**Country of residence**	**2634**		**5846**							
Thailand	269	54.1	229	45.9	Ref			Ref		
Hong Kong	256	46.1	299	53.9	**0.011**	**1.37**	**1.08-1.75**	**<0.001**	**1.93**	**1.37-2.73**
Indonesia	223	41.2	318	58.8	**<0.001**	**1.68**	**1.31-2.14**	0.071	1.44	0.97-2.15
Oman	180	41.2	257	58.8	**<0.001**	**1.68**	**1.30-2.17**	**<0.001**	**2.20**	**1.50-3.25**
Nepal	119	38.3	192	61.7	**<0.001**	**1.90**	**1.42-2.52**	0.253	1.28	0.84-1.95
Malaysia	273	37.9	447	62.1	**<0.001**	**1.92**	**1.53-2.42**	Not included in multivariate model		
Australia	203	37.5	339	62.5	**<0.001**	**1.96**	**1.53-2.51**	Not included in multivariate model		
Libya	38	33.3	76	66.7	**<0.001**	**2.35**	**1.53-3.60**	**<0.001**	**3.54**	**1.91-6.56**
Kuwait	132	31.6	285	68.4	**<0.001**	**2.54**	**1.93-3.33**	**<0.001**	**3.06**	**2.05-4.58**
Bangladesh	284	30.1	644	69.4	**<0.001**	**2.67**	**2.12-3.31**	Not included in multivariate model		
Pakistan	121	28.9	297	71.1	**<0.001**	**2.88**	**2.19-3.80**	0.105	1.40	0.93-2.11
Saudi Arabia	225	28	578	71.9	**<0.001**	**3.02**	**2.38-3.81**	**<0.001**	**2.82**	**1.99-4.01**
UAE	89	21.3	328	78.6	**<0.001**	**4.32**	**3.23-5.80**	**<0.001**	**3.68**	**2.31-5.86**
Jordan	80	14.9	458	85.1	**<0.001**	**6.72**	**5.01-9.04**	**<0.001**	**6.83**	**4.05-11.5**
Syria	53	13	355	87.0	**<0.001**	**7.87**	**5.61-11.0**	**<0.001**	**6.05**	**3.59-10.2**
Palestine	50	12	367	88.0	**<0.001**	**8.62**	**6.11-12.2**	**<0.001**	**4.80**	**2.87-8.02**
Egypt	39	9.4	377	90.6	**<0.001**	**11.4**	**7.81-16.5**	**<0.001**	**9.43**	**5.33-16.7**
**Characteristics**	**FCV-19S Score**				**Unadjusted analyses**			**Adjusted analyses**		
	**Low (score 7-21)**		**High (score 22-35)**							
	**n**	**%**	**n**	**%**	**p**	**ORs**	**95% CIs**	**p**	**AORs**	**95% CIs**
**Country of residence**	**6420**		**2066**							
Libya	104	91.2	10	8.8	Ref			Ref		
Saudi Arabia	714	88.9	89	11.1	0.458	1.30	0.65-2.57	0.669	0.85	0.40-1.82
Thailand	427	85.7	71	14.3	0.123	1.73	0.86-3.46	0.937	1.03	0.47-2.28
Kuwait	347	83.2	70	16.8	**0.037**	**2.1**	**1.04-4.22**	0.395	1.40	0.64-3.07
Oman	351	80.3	86	19.7	**0.008**	**2.55**	**1.28-5.08**	**0.044**	**2.23**	**1.02-4.88**
Jordan	429	79.7	109	20.3	**0.005**	**2.64**	**1.34-5.23**	0.477	0.74	0.33-1.70
Nepal	248	79.7	63	20.3	**0.007**	**2.64**	**1.31-5.35**	0.057	2.16	0.98-4.80
Syria	324	79.6	83	20.4	**0.006**	**2.67**	**1.33-5.32**	0.455	1.35	0.62-2.93
Palestine	330	79.1	87	20.8	**0.004**	**2.74**	**1.37-5.47**	0.844	1.09	0.49-2.42
UAE	320	76.7	97	23.3	**0.001**	**3.15**	**1.59-6.27**	0.561	1.27	0.58-2.81
Indonesia	405	74.8	136	25.1	**<0.001**	**3.50**	**1.77-6.88**	**0.006**	**2.86**	**1.35-6.08**
Malaysia	525	72.9	195	27.1	**<0.001**	**3.87**	**1.98-7.54**	Not included in multivariate model		
Egypt	288	69.2	128	30.8	**<0.001**	**4.62**	**2.34-9.14**	0.055	2.13	0.98-4.62
Hong Kong	382	68.8	173	31.2	**<0.001**	**4.71**	**2.40-9.24**	**0.003**	**3.21**	**1.47-7.01**
Australia	374	68.1	175	31.8	**<0.001**	**4.87**	**2.49-9.54**	Not included in multivariate model		
Pakistan	281	67.2	137	32.8	**<0.001**	**5.07**	**2.57-10.0**	**0.002**	**3.41**	**1.58-7.33**
Bangladesh	571	61.5	357	38.4	**<0.001**	**6.50**	**3.35-12.6**	Not included in multivariate model		
**Characteristics**	**BRCS Score**				**Unadjusted analyses**			**Adjusted analyses**		
	**Low (score 4-13)**		**Medium to High (score 14-20)**							
	**n**	**%**	**n**	**%**	**p**	**ORs**	**95% CIs**	**p**	**AORs**	**95% CIs**
**Country of residence**	**3665**		**4815**							
Australia	534	97.3	15	2.7	Ref			Ref		
Libya	70	61.9	43	38.1	**<0.001**	**21.86**	**11.6-41.4**	Not included in multivariate model		
Pakistan	221	52.8	197	47.1	**<0.001**	**31.73**	**18.3-54.9**	0.210	1.40	0.83-2.36
Jordan	252	46.8	286	53.2	**<0.001**	**40.40**	**23.5-69.4**	**0.014**	**1.99**	**1.15-3.43**
Egypt	191	45.9	225	54.1	**<0.001**	**41.93**	**24.2-72.6**	**0.003**	**2.28**	**1.33-3.88**
Saudi Arabia	354	44.1	448	55.8	**<0.001**	**45.05**	**26.5-76.7**	**0.016**	**1.84**	**1.12-3.02**
Kuwait	183	43.8	234	56.1	**<0.001**	**45.52**	**26.3-78.8**	**0.009**	**2.01**	**1.20-3.40**
Bangladesh	398	42.8	530	57.1	**<0.001**	**47.41**	**27.9-80.5**	Not included in multivariate model		
Hong Kong	230	41.4	325	58.5	**<0.001**	**50.30**	**29.3-86.3**	**0.002**	**2.29**	**1.34-3.91**
UAE	151	36.5	262	63.4	**<0.001**	**61.77**	**35.6-107**	**<0.001**	**2.64**	**1.53-4.55**
Palestine	152	36.5	264	63.4	**<0.001**	**61.83**	**35.7-107**	**<0.001**	**2.90**	**1.68-4.99**
Thailand	175	35.1	323	64.9	**<0.001**	**65.71**	**38.1-113**	**0.004**	**2.18**	**1.29-3.70**
Malaysia	251	34.8	469	65.1	**<0.001**	**66.52**	**38.9-114**	Not included in multivariate model		
Oman	137	31.4	300	68.7	**<0.001**	**77.96**	**44.9-135**	**<0.001**	**3.80**	**2.21-6.54**
Nepal	97	31.2	214	68.8	**<0.001**	**78.54**	**44.6-138**	**<0.001**	**3.45**	**1.99-5.98**
Indonesia	156	28.8	385	71.2	**<0.001**	**87.86**	**50.9-152**	**<0.001**	**4.16**	**2.51-6.92**
Syria	113	27.7	295	72.3	**<0.001**	**92.93**	**53.2-162**	**<0.001**	**4.94**	**2.89-8.46**

*Adjusted for: age, gender, smoking, alcohol intake, living status, place of birth, country, education, employment status, employment stress, healthcare worker, financial impact, contact with COVID-19 case, experience due to COVID-19 and self-identification as a patient*

## Discussion

To our knowledge, this study is one of the few large-scale global cross-sectional studies that assessed psychological distress, levels of fear, and coping strategies and their associated factors among community members, frontline workers, and patients across 17 countries during the first and second wave of the COVID-19 pandemic. We found that more than two-thirds (69%) participants experienced moderate to very high levels of psychological distress and about a quarter (24%) had a high level of fear of COVID-19. Despite having moderate to high levels of psychological distress and fear, more than half of the participants (57%) reported medium to high levels of resilient coping.

Findings from this study were consistent with the previous Australian study [9]. Similarly, the previous research found almost a third of the participants (33%) experienced high to very high levels of psychological distress; however, they found more participants experienced a high level of fear of COVID-19 (32%), while our study found only 24%. Furthermore, the Australian study found that almost all participants (97%) had low resilient coping, whereas this global study found 57% participants had medium to high resilient coping. Learning from previous successful experiences that enable people to cope better could explain this discrepancy [17]. When participants from the Australian study were faced with COVID-19 at an earlier stage, participants of this study (that included participants who were confronted with both 1^st^ and 2^nd^ waves) might have learned how to cope with all kinds of relevant practices from the 1^st^ wave of the pandemic (such as social distancing, home quarantine, or lockdown, hand hygiene and wearing masks), leading them to high resilient coping and less fear of COVID-19. However, the context was interplayed with distress and fear in this study. It was found that participants who perceived distress due to change of their employment, whose financial situation was affected greatly, and had unsure contact with COVID-19 were more likely to have higher psychological distress and fear.

We found that females had higher psychological distress and fear of COVID-19. This finding is consistent with the Australian study, [9] and studies from elsewhere [18]. They also had a greater chance of loneliness, specifically for young people aged 18-29 years or those 60+ [19]. Such distress and fear could also be related to ‘infodemic’ through the increased use of social media [20]. Having a history of mental illness and experience of family violence was shown to aggravate depression, anxiety and stress amongst women during the pandemic [21]. In addition, concerns of exposure to COVID-19 amongst family members could have accentuated their anxiety and distress. Women tend to have more care giving roles in a family and often prioritise health concerns of family members over their own [9]. That warrants improved awareness amongst women regarding regular health assessment and accessing resources to support their wellbeing.

Interestingly, participants who perceived their mental health as good to excellent, even though their financial situation was impacted negatively, and who had contact with COVID-19 patients indirectly or directly were more likely to have medium to high resilient coping. This was especially true for participants who self-identified themselves as nurses. This is incongruent with the Australian study, though consistent with earlier studies [22]. Our findings reflected that participants perceived mental resiliency could be the internal psychological aid that eases their reality during the pandemic despite having higher psychological distress. Enhancing resilience could be a possible intervention to enable people to cope with the mental health impact of COVID-19. Such a psychological resilience model has been developed and tested for its effectiveness in China and was found to improve the overall mental health of the target population during the COVID-19 pandemic [23].

In our study, doctors had higher psychological distress, but low levels of fear of COVID-19; nurses had medium to high resilient coping. A recent systematic review of 24 studies with 13,731 health and social care workers showed that female nurses, comorbidities, lack of personal protective equipment, concerns about family, fear of infections and close contact with COVID-19 patients were the predictors for poor mental wellbeing amongst healthcare workers [24]. Low levels of fear amongst the frontline healthcare workers in our study were likely due to their prolonged professional exposure with COVID-19 patient management. Due to the heterogeneity of the health systems and varying availability of resources across participating countries, healthcare workers experienced catastrophic situations during the surge of pandemic period, which could have resulted in high resilience amongst the nurses.

Our findings showed that participants who had comorbidities and those who had a mental illness showed higher psychological distress and fear. These groups were more vulnerable under pandemic guidelines (such as social distancing, working from home), which potentially raised the risks of relapse, especially those who were mentally ill and who needed primary caregivers. Generally, evidence from clinical settings and literature indicated that mentally ill persons who lived alone would have more psychotic relapses than those being cared for by primary caregivers [25]. Medication adherence for this group of patients could have been challenging without caregiving provision [26]. Accessibility to the health care system was more difficult because most healthcare workers were overloaded with COVID-19 infected patients and the related tasks, therefore, managing chronic diseases was not a priority. In addition, lockdown policies impacted transportation and public facilities were closed in many instances. Previous evidence also suggested that people with stressful situations and pre-existing medical problems had higher levels of depression and anxiety [27]. Telemedicine to replace face-to-face consultations had been established in many countries including Australia during COVID-19. The effect of such an alternative healthcare delivery system needs to be evaluated further, especially its impact on people with non-communicable diseases and/or mental illness who need continuing care.

Eighty-one percent of the study population were never smokers. Those who smoked and drank alcohol, reported increased use of tobacco and alcohol (47% and 30% respectively) in the last six months. Moreover, drinking behavior was also associated with higher levels of fear of COVID-19. The findings were consistent with the previous Australian study and that risky behavior was associated with a higher impact on psychological distress [16]. A study conducted in China also found that participants who had a history of smoking could escalate the severe symptoms of COVID-19 once hospitalized and possibly required ventilator equipment [28]. A Polish study also revealed that current alcohol drinkers were less able to find positives about the pandemic (positive reframing) and coping [29]. An effective coping strategy needs to be developed and implemented to target populations using social media to prevent unhealthy coping behaviors.

The change of employment status and an uncertain financial situation were associated with higher psychological distress and fear. In our study, 51% participants reported that their jobs were affected by COVID-19, due to losing jobs, reduced working hours, or being afraid of job loss. That was probably one of the significant indicators of mental wellbeing, impacted by COVID-19 on people's socioeconomic status around the globe and consistent with a study conducted among Israeli youths (20-35 years old) [30]. The need for urgent action to support and elevate economic assistance, especially for those whose job was impacted negatively from the pandemic, is critical. While business enterprises were freezing around the globe due to restrictions related to controlling the spread of coronavirus, basic needs are essential, specifically for vulnerable groups to prevent psychological crisis which could potentially lead to suicidal attempts or even suicide.

The impact of COVID-19 on the psychological wellbeing was unprecedented and was different from country to country. Therefore, findings from 17 countries were found to be diverse. In our study, country specific results on psychological distress showed a specific trend. For example, more than two-thirds of the participants reported moderate to very high level of psychological distress who were living in countries with war/conflict (Syria, Palestine, Libya and the Middle East [Saudi Arabia, UAE, Jordan and Kuwait]) followed by South Asia (Pakistan, Nepal and Bangladesh) and least by the participants from South-East Asian countries (Thailand, Hong Kong and Indonesia). However, participants from Oman, Australia and Egypt could not be fitted into any of those categories. It can be assumed that such disparities could be related to geography, access to healthcare, having comorbidities, living in war-torn and conflicting countries [31]. It can be also assumed that uncertainties about COVID-19, its progression and rapid mutation, availability and access to varied range of evidence could also contribute to the report of diverse country-wise findings of moderate to high level of psychological distress. Similar higher levels of anxiety were reported in Hong Kong during the SARS epidemic amongst medicine students and students living in the area where there was a rapid spread of infection [32].

Participants from the Middle East and war-torn countries reported less fear compared to the participants from South-East Asian countries and South Asia. The exact reasons for this could not be elicited from our study, however the reasons can be explained by two factors, firstly, high standard care and public health in Saudi Arabia, Kuwait and Oman, and success of early interventions, such as early lockdown reducing the transmission of COVID-19. It can be further emphasized that participants from war-torn countries already have experienced high levels of fear for prolonged periods which might cause an idiosyncratic response to the pandemic [33]. Further study on war-torn counties could provide more insights. Higher levels of fear of COVID-19 among participants from South-East Asian countries could be explained by their previous traumatic experience from SARS and H1N1 pandemics, which disproportionately affected South-East Asian countries [32].

In our study, we found that more than half of the participants (57%) showed medium to high resilience towards the pandemic. Interestingly, participants from Australia found to struggle most, despite reports of very low levels of community transmission compared to the other 16 countries included in this study. This could be explained by the fact that Australian participants were predominantly from Victoria, the only state in Australia which was affected by the second wave of COVID-19 during the study period, which caused statewide strict lockdown, social isolation, job loss [16]. Nonetheless, despite potential lack of capacity and resources to manage pandemics, participants from war-torn countries like Palestine and Syria were found to have higher coping compared to the participants from Australia. It was beyond the scope of our study to examine the reasons for such findings. Research from Syria reported strategies to contain COVID-19, such as effective use of social media tools, community engagement, bottom-up approach from the local government, and coordinated support by the international donor communities [34].

### Limitations

We had some limitations in our study. The use of online surveys potentially introduced selection bias, as participants were limited to those who could access the internet only; therefore, the generalizability of the findings needs to be interpreted with caution. Drawing predictive conclusions based on the differences is difficult and is a limitation of a cross sectional study design. Nevertheless, under the circumstances of movement restriction and social distancing, an online survey was the most robust available option during the pandemic to fulfill our research objectives. From the perspective of multi-country study (17 countries), the multicultural background, the difference of policies and compliance of public health actions that varied across participating countries, might also impact on the examined variables (psychological distress, fear, and ways of coping). We, therefore, adjusted the variable ‘country’ during the multivariate analyses to control potential confounding effects. Furthermore, the collaboration from researchers across 17 countries and the achievement of the target sample size during the crisis period of COVID-19 showed significant power to test our hypotheses and provided key information to plan interventions as needed.

## Conclusions

Our study examined the extent and identified factors associated with psychological distress, fear of COVID-19 and coping amongst diverse community members across 17 countries. Females and people with existing mental health issues were the most vulnerable group of populations for adverse psychological impact of COVID-19. There is an urgent need to prioritise these vulnerable population; adequate medical and social support along with specific health promotion policies should be considered within the strategic response to the ongoing pandemic and future crises. Future studies should focus on developing strategies to enhance resilience and examining effectiveness of such interventions. Besides global strategies to address psychological impact, policy makers in each country should revisit existing support structures and enhance them during this critical period. Innovative approaches are needed to enhance effective coping and social support to alleviate impact and prevent emotional crisis for vulnerable people in the longer term.

## Supplementary Information

**Additional file 1: Table S1.** Levels of psychological distress among the study participants (based on K-10 scoring). **Table S2.** Levels of fear of COVID-19 among the study participants (based on the FCV-19S scoring). **Table S3.** Coping during COVID-19 pandemic among the study participants.

## Data Availability

All data generated or analysed during this study are included in this published article.
