# Supplementary material for: COVID-19: Factors associated with psychological distress, fear, and coping strategies among community members across 17 countries
Source: Global Health. 2021 Oct 1;17:117. doi: 10.1186/s12992-021-00768-3 (PMC8485312; doi:10.1186/s12992-021-00768-3)
Supplement: Supplementary file 1 — Additional file 1: Table S1. Levels of psychological distress among the study participants (based on K-10 scoring). Table S2. Levels of fear of COVID-19 among the study participants (based on the FCV-19S scoring). Table S3. Coping during COVID-19 pandemic among the study participants. [file 12992_2021_768_MOESM1_ESM.docx]

**SUPPLEMENTARY**

***Table S.1: Levels of psychological distress among the study participants (based on K-10 scoring)***

| **K-10 items** | **Total, n(%)** |
| --- | --- |
| **About how often did you feel tired out for no good reason?** | **8484** |
| None | 2069 (24.4) |
| A little of the time | 2210 (26.0) |
| Some of the time | 2815 (33.2) |
| Most of the time | 1107 (13.0) |
| All of the time | 283 (3.3) |
| **About how often did you feel nervous?** | **8485** |
| None | 1845 (21.7) |
| A little of the time | 2586 (30.5) |
| Some of the time | 2567 (30.3) |
| Most of the time | 1091 (12.9) |
| All of the time | 396 (4.7) |
| **About how often did you feel so nervous that nothing could calm you down?** | **8484** |
| None | 3949 (46.5) |
| A little of the time | 2063 (24.3) |
| Some of the time | 1661 (19.6) |
| Most of the time | 592 (7.0) |
| All of the time | 219 (2.6) |
| **About how often did you feel hopeless?** | **8485** |
| None | 3588 (42.3) |
| A little of the time | 2195 (25.9) |
| Some of the time | 1679 (19.8) |
| Most of the time | 740 (8.7) |
| All of the time | 283 (3.3) |
| **About how often did you feel restless or fidgety?** | **8485** |
| None | 2554 (30.1) |
| A little of the time | 2454 (28.9) |
| Some of the time | 2258 (26.6) |
| Most of the time | 960 (11.3) |
| All of the time | 259 (3.1) |
| **About how often did you feel so restless you could not sit still?** | **8485** |
| None | 4197 (49.5) |
| A little of the time | 2141 (25.2) |
| Some of the time | 1493 (17.6) |
| Most of the time | 508 (6.0) |
| All of the time | 146 (1.7) |
| **About how often did you feel so depressed?** | **8484** |
| None | 3148 (37.1) |
| A little of the time | 2469 (29.1) |
| Some of the time | 1830 (21.6) |
| Most of the time | 746 (8.8) |
| All of the time | 291 (3.4) |
| **About how often did you feel that everything was an effort?** | **8484** |
| None | 2245 (26.5) |
| A little of the time | 2457 (29.0) |
| Some of the time | 2173 (25.6) |
| Most of the time | 1113 (13.1) |
| All of the time | 496 (5.8) |
| **About how often did you feel so sad that nothing could cheer you up?** | **8484** |
| None | 3518 (41.5) |
| A little of the time | 2207 (26.0) |
| Some of the time | 1709 (20.1) |
| Most of the time | 732 (8.6) |
| All of the time | 318 (3.7) |
| **About how often did you feel worthless?** | **8485** |
| None | 4438 (52.3) |
| A little of the time | 1862 (21.9) |
| Some of the time | 1280 (15.1) |
| Most of the time | 552 (6.5) |
| All of the time | 353 (4.2) |
| **K10 score (total)** | **8480** |
| Mean (±SD) | 21.6 (8.9) |
| Range | 10 to 50 |
| **Level of psychological distress (K10 categories)** | **8480** |
| Low (score 10-15) | 2634 (31.1) |
| Moderate (score 16-21) | 2173 (25.6) |
| High (score 22-29) | 1964 (23.2) |
| Very high (score 30-50) | 1709 (20.2) |

***Table S.2: Levels of fear of COVID-19 among the study participants (based on the FCV-19S scoring)***

| **FCV-19S items** | **Total, n(%)** |
| --- | --- |
| **I am most afraid of COVID-19** | **8486** |
| Strongly disagree | 1529 (18.0) |
| Disagree | 1557 (18.3) |
| Neither agree nor disagree | 2072 (24.4) |
| Agree | 2454 (28.9) |
| Strongly agree | 874 (10.3) |
| **It makes me uncomfortable to think about COVID-19** | **8486** |
| Strongly disagree | 1588 (18.7) |
| Disagree | 1581 (18.6) |
| Neither agree nor disagree | 1771 (20.9) |
| Agree | 2782 (32.8) |
| Strongly agree | 764 (9.0) |
| **My hands become clammy when I think about COVID-19** | **8486** |
| Strongly disagree | 4456 (52.5) |
| Disagree | 1917 (22.6) |
| Neither agree nor disagree | 1240 (14.6) |
| Agree | 677 (8.0) |
| Strongly agree | 196 (2.3) |
| **I am afraid of losing my life because of COVID-19** | **8486** |
| Strongly disagree | 2753 (32.4) |
| Disagree | 1606 (18.9) |
| Neither agree nor disagree | 1688 (19.9) |
| Agree | 1744 (20.6) |
| Strongly agree | 695 (8.2) |
| **When watching news and stories about COVID-19 on social media, I become nervous or anxious** | **8486** |
| Strongly disagree | 1901 (22.4) |
| Disagree | 1475 (17.4) |
| Neither agree nor disagree | 1711 (20.2) |
| Agree | 2706 (31.9) |
| Strongly agree | 693 (8.2) |
| **I cannot sleep because I’m worrying about getting COVID-19** | **8486** |
| Strongly disagree | 4745 (55.9) |
| Disagree | 1756 (20.7) |
| Neither agree nor disagree | 1198 (14.1) |
| Agree | 639 (7.5) |
| Strongly agree | 148 (1.7) |
| **My heart races or palpitates when I think about getting COVID-19** | **8486** |
| Strongly disagree | 4176 (49.2) |
| Disagree | 1727 (20.4) |
| Neither agree nor disagree | 1283 (15.1) |
| Agree | 1058 (12.5) |
| Strongly agree | 242 (2.9) |
| **FCV-19S score (total)** | **8486** |
| Mean (±SD) | 16.9 (6.5) |
| Range | 7 to 35 |
| **Level of fear of COVID-19 (FCV-19S categories)** | **8486** |
| Low (score 7-21) | 6420 (75.7) |
| High (score 22-35) | 2066 (24.3) |

***Table S.3: Coping during COVID-19 pandemic among the study participants***

| **BRCS items** | **Total, n(%)** |
| --- | --- |
| **I look for creative ways to alter difficult situations** | **8480** |
| Does not describe me at all | 656 (7.7) |
| Does not describe me | 1040 (12.3) |
| Neutral | 3056 (36.0) |
| Describes me | 2899 (34.2) |
| Describes me very well | 829 (9.8) |
| **Regardless of what happens to me, I believe I can control my reaction to it** | **8480** |
| Does not describe me at all | 447 (5.3) |
| Does not describe me | 1017 (12.0) |
| Neutral | 2583 (30.5) |
| Describes me | 3426 (40.4) |
| Describes me very well | 1007 (11.9) |
| **I believe I can grow in positive ways by dealing with difficult situations** | **8480** |
| Does not describe me at all | 434 (5.1) |
| Does not describe me | 682 (8.0) |
| Neutral | 2116 (25.0) |
| Describes me | 3908 (46.1) |
| Describes me very well | 1340 (15.8) |
| **I actively look for ways to replace the losses I encounter in life** | **8480** |
| Does not describe me at all | 484 (5.7) |
| Does not describe me | 901 (10.6) |
| Neutral | 2736 (32.3) |
| Describes me | 3293 (38.8) |
| Describes me very well | 1066 (12.6) |
| **BRCS score (total)** | **8480** |
| Mean (±SD) | 13.7 (3.3) |
| Range | 4 to 20 |
| **Level of coping (BRCS categories)** | **8480** |
| Low resilient copers (score 4-13) | 3665 (43.2) |
| Medium resilient copers (score 14-16) | 3479 (41.0) |
| High resilient copers (score 17-20) | 1336 (15.8) |
